# Gastroenteropancreatic neuroendocrine tumour arising in Meckel’s diverticulum coexisting with colon adenocarcinoma

**DOI:** 10.1186/1477-7819-12-358

**Published:** 2014-11-27

**Authors:** Darko Katalinic, Fedor Santek, Antonio Juretic, Dinko Skegro, Stjepko Plestina

**Affiliations:** Department of Oncology, University Hospital Centre Zagreb, University of Zagreb School of Medicine, Kispaticeva 12, HR-10000 Zagreb, Croatia; Department of Internal Medicine, University Hospital Merkur, University of Zagreb School of Medicine, Zajceva 19, HR-10000 Zagreb, Croatia

**Keywords:** Colon adenocarcinoma, Gastroenteropancreatic neuroendocrine tumour, Meckel’s diverticulum

## Abstract

Although colon cancer is the third most common cause of cancer-related death worldwide, the prevalence of gastroenteropancreatic neuroendocrine tumours (GEP-NETs) remains rare. To date, very few cases of GEP-NETs within Meckel’s diverticulum and synchronous colorectal cancer have been reported. Although the coexistence of these two tumour types is uncommon, it is important to be aware of their disease patterns. We present a rare case of a patient with an intestinal GEP-NET arising in Meckel’s diverticulum coexisting with metastatic colon adenocarcinoma, and we discuss the clinical manifestations and the diagnostic procedures and treatment modalities used. This case report underlines the importance of being aware of this particular coexistence, as well as the unlikely metastatic spread of GEP-NETs and the importance of a multidisciplinary approach to cancer treatment. Finally, individualizing the treatment according to the stages of the primaries will result in durable cancer control, particularly in synchronous double malignancy.

## Background

Neuroendocrine gastroenteropancreatic tumours (GEP-NETs) constitute a heterogeneous group of genetically diverse neoplasms arising from the secretory cells of the neuroendocrine system, with the primary tumours located in the digestive tract[1]. Their incidence is now estimated to be 2 to 5/100,000/year, but this is probably an underestimate. Most commonly, the primary lesion is located in the gastric mucosa, the small and large intestines, the rectum and the pancreas[2]. The development of a second primary malignancy in patients with these tumours is uncommon, but it has been described. However, coexistence of colon adenocarcinoma and intestinal GEP-NET arising in a Meckel’s diverticulum is very rare and represents diagnostic and therapeutic challenge[3]. We report a case of a patient with GEP-NET in Meckel’s diverticulum synchronous with metastatic colon cancer, all of which were resected during the same laparotomy, and discuss the clinical manifestations as well as the diagnostic procedures and treatment modalities used.

## Case presentation

**Figure 1:**
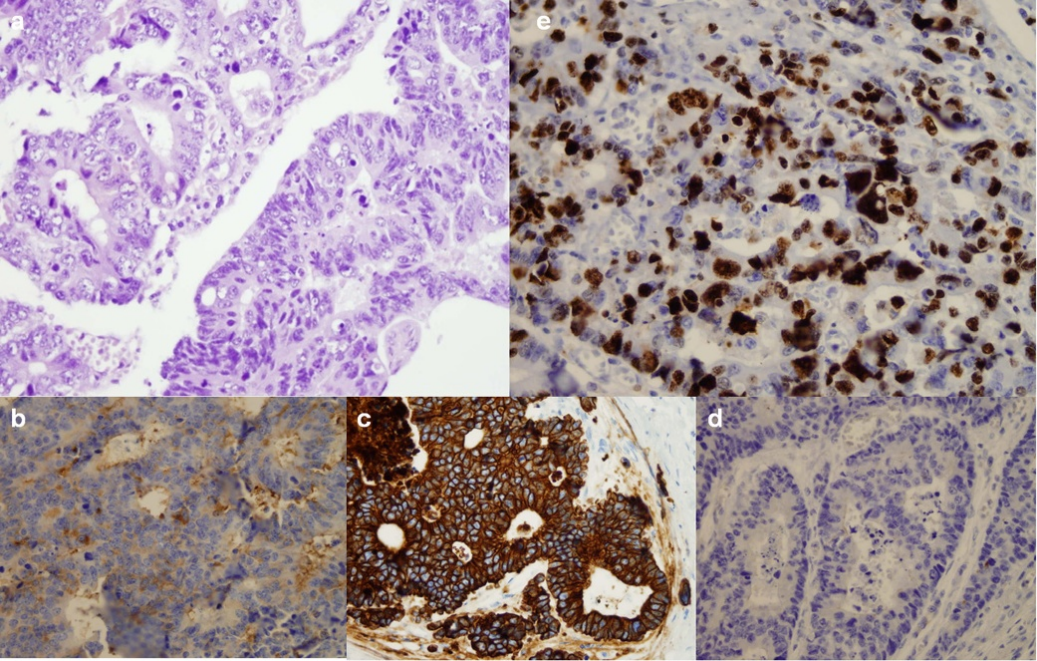
**Histological and immunohistochemical stains of colon adenocarcinoma. (a)** Solid tumour nests and large polygonal cells (haematoxylin and eosin stain; original magnification, ×400). Immunohistochemical stains reveal positive findings for synaptophysin **(b)** (original magnification, ×400), cytokeratin AE1/AE3 **(c)** (original magnification, ×400) and chromogranin A **(d)** (original magnification, ×400). **(e)** Antigen Ki-67 immunostaging was positive in 60% of tumour cells (original magnification, ×400).

**Figure 2:**
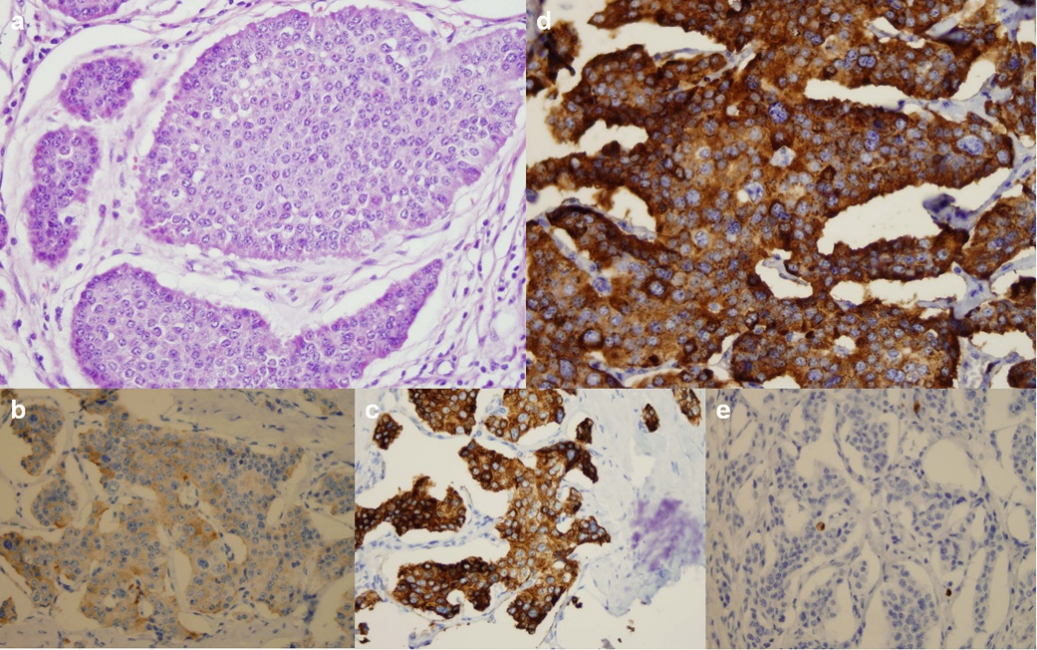
**Histological and immunohistochemical stains of Meckel’s diverticulum. (a)** Moderately differentiated neuroendocrine tumour (haematoxylin and eosin stain; original magnification, ×400). Tumour cells show cytoplasmic staining for synaptophysin **(b)** (original magnification, ×400), cytokeratin AE1/AE3 **(c)** (original magnification, ×400) and chromogranin A **(d)** (original magnification, ×400). **(e)** Antigen Ki-67 immunostaging was positive in 3% of tumour cells. (original magnification, ×400).

**Figure 3:**
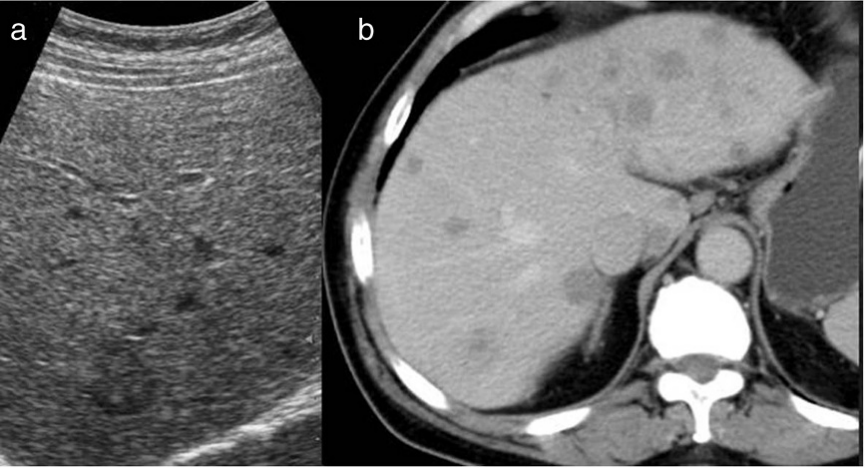
**Radiological evaluation of liver metastases. (a)** Ultrasound. **(b)** Unenhanced computed tomographic scan.

A 63-year-old man presented at our hospital with abdominal distension and diarrhoea 1 month before hospital admission. Apart from elevated serum γ-glutamyltranspeptidase (233 U/L; normal range, 9 to 35 U/L), carcinoembryonic antigen (CEA) (89.3 ng/ml; range, 0 to 5 ng/ml), carbohydrate antigen 19-9 (CA 19-9) (2,052 U/ml; range, 0 to 37 U/ml) and hepatomegaly, all other systems and laboratory findings were normal. A colonoscopy revealed intraluminal stenosis with a reddish, irregularly shaped mass located in the ascending colon, 15.5 cm from the ileocaecal valve, so biopsy specimens were obtained. An open right hemicolectomy with ileotransverse anastomosis was performed. Intraoperatively, the interior surface of the colon demonstrated an invasive, exophytic, crater-like tumour. The clinical and pathological information about the patient is summarized in Table 1. A microscopic examination showed an infiltrating tumour of the ascending colon, measuring 3.5 × 3.0 × 2.6 cm with extension into the pericolic fat and mesenteric lymphatics (involving 10 of 23 regional lymph nodes), with neural and vascular invasion. Histological sections revealed a connection to the intestinal mucosa with extension into the underlying muscularis propria, but without serosal affection. The tubular resection margins were without tumour involvement. The immunohistochemical stains (cytokeratin AE1/AE3 (CKAE1/AE3)–positive, chromogranin A (CgA)–positive, synaptophysin-positive, antigen Ki-67 (Ki-67) at 60%, with >20 mitoses/10 high-power fields (hpf)) supported the diagnosis of a metastatic, poorly differentiated form of high-grade colon adenocarcinoma (grade G3, pT3pN2bpM1a, Astler-Coller classification D[4], American Joint Committee on Cancer (AJCC) stage IVA[5]) (Figure 1). Genetic testing for mutations in codons 12 or 13 of the *KRAS* gene was negative. Moreover, a mass in Meckel’s diverticulum, 8.7 cm proximal to the ileocaecal valve, was encountered. The histological examination revealed a firm, grey-white tumour composed of neoplastic neuroendocrine cells in the submucosa arranged in a nesting pattern measuring 3 × 2 × 2 cm. There was penetration of the muscular layer and lymphatic vessels, but without serosal, neural or vascular invasion. The resection margins were free of tumour. Immunohistochemical staging confirmed a well-differentiated, low-grade GEP-NET (low grade (grade G1), pT3pN0pM0, AJCC stage IIB, CKAE1/AE3–positive, CgA–positive, synaptophysin-positive, Ki-67 at 3%, with 2 mitoses/10 hpf, without necrosis) (Figure 2) according to World Health Organisation and European Neuroendocrine Tumour Society guidelines[6]. There was evidence of multiple hepatic metastases from colon carcinoma, based on abdominal ultrasonography, computed tomography (CT) (Figure 3) and liver biopsy specimens. The postoperative indium-111-diethylenetriaminepentaacetic acid-D-phenylalanine-octreotide scintigraphy result was negative.

**Table 1:** **Clinical and pathological data of the patient** ^**a**^

	Colon adenocarcinoma	Neuroendocrine tumour
Tumour size	3.5 × 3.0 × 2.6 cm	3 × 2 × 2 cm
Fat tissue invasion	+	-
Lymph node invasion	+ (10/23)	- (0/23)
Perineural invasion	+	-
Vascular invasion	+	-
Lymphatic vessel invasion	+	+
Muscularis propria invasion	+	+
Serosal invasion	+	-
Resection margins	-	-
Tumour necrosis	-	-
CKAE1/AE3	+	+
CgA	+	+
*KRAS*	-	Not applicable
Synaptophysin	+	+
Ki-67	60%	3%
Mitoses/10 hpf	>20	≤2
Grade of the cancer	High grade (G3)	Low grade (G1)
Astler-Coller classification[4]	D	Not applicable
TNM stage	pT3pN2bpM1a	pT3pN0pM0
AJCC clinical stage[5]	IVA	IIB

^a^AJCC, American Joint Committee on Cancer; CgA, Chromogranin A; CKAE1/AE3, Cytokeratin AE1/AE3; hpf, High-power fields; Ki-67, Antigen Ki-67; *KRAS*, v-Ki-ras2 Kirsten rat sarcoma viral oncogene homolog; TNM, Tumour, node, metastasis classification of malignant tumours.

The patient was seen by a medical oncologist with a view to starting chemotherapy. Because there was only evidence of colon cancer metastases, we decided to start with a FOLFIRI (folinic acid, fluorouracil, irinotecan) chemotherapy regimen, which has been reported to induce major tumour responses in 40% to 50% of patients with stage IV colon cancer[7–9]. Our patient has received 22 cycles of chemotherapy as of this writing, and he tolerates the treatment very well. He was in an excellent general condition with good performance status and without any sign of tumour progression or additional metastases 11 months after the diagnosis.

## Discussion

Meckel’s diverticulum is a vestigial remnant of the omphalomesenteric duct. It is the most common embryonic malformation of the gastrointestinal tract and is present in approximately 2% of the population, with men experiencing symptoms more frequently than women[10]. Its prevalence is three to five times higher in men than in women. Only 2% of cases are symptomatic, and these usually are children[11]. The condition was first described by Fabricius Hildanus in the 16th century and was later named after Johann F Meckel, who explained the embryologic origin of this entity in 1809[12]. As the omphalomesenteric duct is made up of pluripotent cell lining, Meckel’s diverticulum may harbour embryonic remnants of other tissues. Heterotopic rests of gastric or intestinal mucosa and pancreatic tissue are seen in most cases[11]. The majority of Meckel’s diverticula remain asymptomatic throughout life. In general, they are discovered accidentally during different surgical procedures. The main complications associated with Meckel’s diverticulum are bleeding, ulceration, inflammation, perforation, ileus, intussusception or neoplastic transformation. However, tumours associated with Meckel’s diverticula occur with a frequency of 1% to 5%[13], and most of them are found incidentally during surgery[14]. Most of them are benign tumours, such as adenomas, leiomyomas, angiomas and lipomas. Malignant neoplasms mainly include gastric adenocarcinomas, sarcomas and, rarely, gastrointestinal stromal tumours and different types of NETs[3].

Generally, the NETs arise from the dispersed endocrine system and may originate from almost any location in the human body. They are most commonly found in the gastrointestinal tract (such as GEP-NETs) and the respiratory system. As neuroendocrine phenotypes and genetics have become better understood, the definition of neuroendocrine cells has changed and is now accepted as referring to cells with neuromodulator, neuropeptide or neurotransmitter hormone production; dense-core secretory granules; and absence of axons and synapses. GEP-NETs have attracted much attention in recent years because they are often slow-growing neoplasms which are relatively easy to palliate. Moreover, pharmacological therapy has dramatically improved symptom control, thus offering different targeted therapies for metastatic or inoperable disease[1]. This kind of rare cancer is usually diagnosed because of symptoms related to the overproduction of hormones by the tumours, and/or because of complications related to the presence of the tumour mass, and/or as an incidental finding during imaging or surgical procedures[15]. Depending on whether secreted hormone is detectable and associated symptoms are present, GEP-NETs can be divided into ‘functioning’ and ‘nonfunctioning’ subtypes. Functioning tumours are slow-growing tumours, and morbidity often results from the secreted hormone (or hormones) rather than the tumour mass. In cases of nonfunctioning tumours, it is accepted that there may be secreted, but as yet undetectable, hormones. These nonfunctioning tumours tend to be more aggressive with symptoms of tumour bulk[1].

In the past, the phenomenon of multiple primary cancers was considered a medical curiosity. As anticipated, multicentric cancers in a single organ, in paired organs or in contiguous tissues are now known to occur with increasing frequency. Generally, they fall into two categories: (1) synchronous, in which the tumours occur at the same time; and (2) metachronous, in which the tumours follow in sequence. In the 1930s, Warren and Gates[16], in a study of 1,078 autopsies of cancer patients, revealed that 40 patients, or 3.7%, had either occult or clinically apparent second primary cancers. The data indicate that, in a person who is genetically predisposed to develop cancer, he or she will more often develop it earlier in life than a person who develops cancer sporadically. The precise pathogenesis of secondary cancers associated with NETs remains unclear and quite complex, with genetic, environmental, hormonal, medical treatment–related and gender-specific factors, as well as interactions thereof, undoubtedly playing roles[17].

According to a previous study, gastrin and cholecystokinin were associated with NETs; this resulted in tissue growth in the gastrointestinal tract and carcinogenesis that led to colorectal and gastric cancers[18]. In addition, other authors have reported that NETs are associated with a high risk of a secondary gastrointestinal malignancy. They studied 96 patients with NETs and found that 14 patients had a NET and a second primary malignancy[19]. In patients known to have NETs, the possibility of a hypersecretion syndrome, such as the paraneoplastic or carcinoid syndrome, always has to be strongly considered. In a study of NETs, excluding carcinoid tumours, Vilallonga *et al*.[20] found that 5 of 2,155 colorectal cancer patients were identified, and all of the patients presented with a paraneoplastic or carcinoid syndrome. However, our patient had no such symptoms. Regardless of this, we cannot rule out the possibility of secreted peptides or hormones in the plasma at very low concentrations not associated with clinical symptoms, or, if there were secreted peptides, they were not associated with clinical effects. As NETs are normally diagnosed after the results of histological evaluation of primary tumours or metastases are obtained, imaging is usually used for disease staging and further therapy planning. CT, magnetic resonance imaging and somatostatin receptor nuclear imaging, using either single-photon emission CT or positron emission tomography (PET) modalities, seems particularly effective in localizing the primary tumour and its metastases[21, 22].

Surgery remains the best method of treatment for NETs, regardless of their localization. It is the preferred option for patients with resectable disease, but palliation with tumour debulking, chemotherapy and targeted radionuclide therapy is often needed. For localized disease, only surgery provides the possibility of complete remission[23, 24]. However, metastases to the regional lymph nodes or distant parenchymal micrometastases may be present at the time of surgery. The effectiveness of adjuvant therapy with chemoradiotherapy in disseminated NETs has not been completely evaluated. In our case, the patient did not have a secretory NET, paraneoplastic symptoms or signs or any ectopic secretion of hormones. However, in patients with somatostatin receptor–positive NETs, somatostatin analogues given subcutaneously or intramuscularly (octreotide LAR given in doses of 10, 20 or 30 mg every 4 weeks as a deep intramuscular injection or lanreotide given in doses of 60, 90 or 120 mg every 4 weeks as a deep subcutaneous injection) alleviate symptoms by blocking hormone release[22, 25]. Chemotherapy is not considered a part of first-line therapy for NETs, because the regimens used to date have not been effective as surgical treatments. Cisplatin used with etoposide, and streptozocin used in combination with doxorubicin and 5-fluorouracil, results in partial tumour response in more than half of patients, as measured by radiologic and serologic testing[26, 27]. Targeted chemotherapy agents (for example, everolimus, sunitinib) have been also used based on improved progression-free survival. Continuous daily administration of sunitinib at a dose of 37.5 mg improved progression-free survival, overall survival and the objective response rate as compared with placebo among patients with advanced NETs. Everolimus, as compared with placebo, was associated with a 6.4-month prolongation of the median progression-free survival[28, 29]. Isolated metastases to the liver can be treated by radiofrequency ablation or hepatic artery embolization, or by transarterial chemoembolization, which combines hepatic artery embolization with hepatic artery chemoinfusion[30]. Selective internal radiation therapy for neuroendocrine metastases to the liver delivers radioactive microsphere therapy by injection directly into the hepatic artery[31]. In selected cases, a radionuclide therapy (yttrium-90- or lutetium-177-labelled analogues, iodine-131-meta-iodobenzylguanidine) may also be considered[32].

The prognosis for, and long-term survival of, patients with NETs has improved with the advent of more aggressive surgical intervention and the use of long-acting somatostatin agonists and targeted second-line therapy. Recent studies have demonstrated that malignant disease, defined by direct invasion of adjacent organs by tumours, lymph node metastases or distant organ spread, may have 5-year survival rates as high as 77% to 95% when treated aggressively with resection of primary tumours and adjunctive therapy[33, 34]. For localized and well-differentiated tumours treated with complete surgical resection, 5-year survival approaches 90% for Meckel’s diverticulum NETs[35]. Favourable prognostic factors include curative resection of the primary tumour, absence of liver metastases and metachronous liver metastases, and aggressive treatment of liver metastases[36]. Unfortunately, nearly all patients with metastatic disease have recurrence by the 7-year follow-up, even after successful treatment[34].

In addition, surgery is the only curative modality for localized colorectal cancer (stages I and II). Chemotherapy is standard management for patients with high-risk stage II, stage III or stage IV disease (capecitabine, 5-fluorouracil, irinotecan, oxaliplatin). Biologic agents (bevacizumab, cetuximab, panitumumab, regorafenib, aflibercept) have assumed a major role in the treatment of metastatic cases, with selection increasingly guided by genetic analysis of the tumour. At present, the role of radiation therapy is limited to palliative therapy for selected metastatic sites, such as bone or brain[37–40].

All patients with synchronous colorectal cancer and GEP-NETs must be extensively evaluated and clinically monitored during the workup and follow-up period in order to detect disease progression or relapse. Typically, patients are seen every 3 to 6 months during the first 3 years and every 6 to 12 months thereafter. Routine laboratory tests, tumour markers (CEA, CA 19-9, CGA, 5-hydroxyindoleacetic acid), endoscopic investigation of the gastrointestinal tract, abdominal ultrasound, ultrasound of lymph nodes, chest radiography and CT or whole-body PET/PET-CT scans may be used during follow-up. We consider that early diagnosis with complete preoperative examination, careful intraoperative exploration, periodic postoperative surveillance and radical resection can increase survival time. The treatment strategy depends on many factors, such as the surgical approach, the patient’s general condition, the tumour grade, the extent of the disease and the patient’s response to therapy. The treatment strategy needs to be individualized for each patient[41, 42]. However, all malignant tumours, because of their genomic instability, have tremendous redundancy in their ability to maintain growth and to spread into surrounding tissues and distant organs. This remarkable level of complexity makes successful treatment of colon cancer coexisting with GEP-NET all the more challenging.

## Conclusions

The occurrence of synchronous primary cancers remains an issue of great interest to surgeons, and oncologists in particular, as well as the field of medicine in general. The question of common genetic pathways in the pathogenesis of such tumours is always raised when such associations are seen. Because multiple primary cancers can no longer be considered rare, the physician should be alert to the possibility of their occurrence. However, NETs arising in a Meckel’s diverticulum represent a significant clinical challenge because they have varied presentations, and initial imaging studies to locate the tumour may be inconclusive. Our case addresses several important aspects of clinical interest. First, only a very small number of cases have been reported with synchronous GEP-NET within Meckel’s diverticulum and colon adenocarcinoma. Second, there are no clear guidelines for appropriate treatment and follow-up of patients with GEP-NET with a synchronous secondary malignancy; therefore, our experience may help in the consideration of treatment for similar patients. The case that we report also emphasizes the importance of a thorough exploration of the abdomen when carrying out elective laparotomies. The impact of synchronously existing cancers on the overall prognosis of a patient must always be considered when planning therapy in such instances as well. Finally, patients who manifest multiple primary cancers represent a unique experiment of nature that may challenge new avenues of investigation into the complex aetiology of tumours.

## Consent

Written informed consent was obtained from the patient for publication of this Case report and any accompanying images. A copy of the written consent is available for review by the Editor-in-Chief of this journal.

## Abbreviations

Ca 19-9: Carbohydrate antigen 19-9
CEA: Carcinoembryonic antigen
CgA: Chromogranin A
CKAE1/AE3: Cytokeratin AE1/AE3
CT: Computed tomography
ENETS: European Neuroendocrine Tumour Society
GEP-NET: Gastroenteropancreatic neuroendocrine tumour
hpf: High-power fields
Ki-67: Antigen Ki-67
KRAS: v-Ki-ras2 Kirsten rat sarcoma viral oncogene homolog
NET: Neuroendocrine tumour
TNM: Tumour, node, metastasis classification of malignant tumours.
